# Accuracy of contrast-enhanced spectral mammography for estimating residual tumor size after neoadjuvant chemotherapy in patients with breast cancer: a feasibility study

**DOI:** 10.1590/0100-3984.2016-0029

**Published:** 2017

**Authors:** Filipe Ramos Barra, Fernanda Freire de Souza, Rosimara Eva Ferreira Almeida Camelo, Andrea Campos de Oliveira Ribeiro, Luciano Farage

**Affiliations:** 1 MD, Radiologist in the Department of Breast Imaging, Imagens Médicas de Brasília - IMEB, Brasília, DF, Brazil.; 2 MD, Professor at the School of Medical Sciences, Universidade de Brasília (UnB), Brasília, DF, Brazil.

**Keywords:** Mammography/methods, Breast neoplasms/diagnosis, Magnetic resonance imaging, Neoadjuvant therapy/methods

## Abstract

**Objective:**

To assess the feasibility of contrast-enhanced spectral mammography (CESM) of the breast for assessing the size of residual tumors after neoadjuvant chemotherapy (NAC).

**Materials and methods:**

In breast cancer patients who underwent NAC between 2011 and 2013, we evaluated residual tumor measurements obtained with CESM and full-field digital mammography (FFDM). We determined the concordance between the methods, as well as their level of agreement with the pathology. Three radiologists analyzed eight CESM and FFDM measurements separately, considering the size of the residual tumor at its largest diameter and correlating it with that determined in the pathological analysis. Interobserver agreement was also evaluated.

**Results:**

The sensitivity, specificity, positive predictive value, and negative predictive value were higher for CESM than for FFDM (83.33%, 100%, 100%, and 66% vs. 50%, 50%, 50%, and 25%, respectively). The CESM measurements showed a strong, consistent correlation with the pathological findings (correlation coefficient = 0.76-0.92; intraclass correlation coefficient = 0.692-0.886). The correlation between the FFDM measurements and the pathological findings was not statistically significant, with questionable consistency (intraclass correlation coefficient = 0.488-0.598). Agreement with the pathological findings was narrower for CESM measurements than for FFDM measurements. Interobserver agreement was higher for CESM than for FFDM (0.94 vs. 0.88).

**Conclusion:**

CESM is a feasible means of evaluating residual tumor size after NAC, showing a good correlation and good agreement with pathological findings. For CESM measurements, the interobserver agreement was excellent.

## Abstract (pt)

**Objetivo:**

Avaliar a viabilidade da utilização da mamografia espectral com meio de contraste (CESM) na avaliação do tumor residual em mulheres com câncer de mama submetidas a quimioterapia neoadjuvante.

**Materiais e métodos:**

Foi avaliada a concordância entre a mensuração do tumor residual na CESM e na mamografia digital (FFDM) com os dados histopatológicos de mulheres submetidas a quimioterapia neoadjuvante entre 2011 e 2013. Após as exclusões, três radiologistas analisaram oito CESMs e FFDMs separadamente. A maior dimensão do tumor residual foi considerada para comparação com os resultados histopatológicos. Concordância e correlação da CESM e FFDM com resultados histopatológicos e a concordância interobservador foram avaliadas.

**Resultados:**

A CESM teve sensibilidade, especificidade e valores preditivos positivos e negativos maiores que a FFDM - 83,33%, 100%, 100% e 66% *versus* 50%, 50%, 50% e 25%, respectivamente. A CESM teve correlação boa e consistente com os achados histopatológicos (coeficiente de correlação = 0,76-0,92; coeficiente de correlação intraclasse = 0,692-0,886). A correlação entre FFDM e os achados histopatológicos não foi estatisticamente significante, com consistência questionável (coeficiente de correlação intraclasse = 0,488-0,598). A concordância entre as dimensões do estudo histopatológico foi mais estreita com a CESM do que com a FFDM. A concordância interobservador foi maior na CESM (0,94) do que na FFDM (0,88).

**Conclusão:**

A CESM é viável e pode ser utilizada para avaliação de tumor residual após quimioterapia neoadjuvante. A CESM tem boa correlação e concordância com o estudo histopatológico e excelente concordância interobservador.

## INTRODUCTION

Neoadjuvant chemotherapy (NAC) is an established component of breast cancer treatment. Some advantages of NAC include a reduction in tumor size, early treatment of micrometastatic disease, and *in vivo* assessment of tumor response^(1,2)^. The accurate assessment of residual tumor extent after NAC is critical for surgical planning. Overestimation of the tumor extent can lead to unnecessary mastectomy, whereas underestimation can increase the risk of positive surgical margins.

Although a complete pathological response is not prognostic for disease-free survival in all breast cancer subtypes, the post-NAC extent of residual disease in the breast and lymph nodes is associated with patient survival^(3)^. Patients with a complete pathological response have a lower risk of locoregional relapse and are candidates for less extensive locoregional treatment^(4)^.

Physical examination, ultrasound, and mammography have been used in order to assess residual tumor size in breast cancer patients after NAC, although the accuracy of these techniques is not satisfactory^(5-7)^. Magnetic resonance imaging (MRI) of the breast is currently the best modality for monitoring tumor response and for assessing residual disease after NAC because it is more accurate than are mammography, ultrasound, and clinical examination^(8,9)^. However, MRI is a time-consuming exam, usually lasting 30-45 min, and requires a dedicated coil, as well as trained readers.

Contrast-enhanced spectral mammography (CESM) is an imaging modality that combines contrast enhancement with digital mammography. Nonionic iodinated contrast, which is administered intravenously, allows lesions to be characterized based on their enhancement. Each CESM exposure is composed of a low-energy image, similar to that obtained with full-field digital mammography (FFDM), and a high-energy image with an X-ray spectrum above the k-edge of iodine (33.2 keV). The two images are recombined, and a subtraction image of the lesions is produced^(10,11)^. Initial studies comparing CESM with mammography, ultrasound, and MRI show that CESM is better at detecting suspicious lesions than are mammography and mammography plus ultrasound, as well as having an accuracy in lesion size measurement similar to that of MRI^(12-16)^.

The purpose of this study was to evaluate the feasibility of using CESM to assess residual tumor extent after NAC in breast cancer patients. Specific objectives were to evaluate the accuracy of CESM in determining residual tumor size, using pathology results as the gold standard, to compare the performance of CESM with that of FFDM (low-energy images only), in terms of their performance, and to analyze interobserver agreement.

## MATERIALS AND METHODS

### Patients and treatment

This was a retrospective study. The study protocol was approved by the local research ethics committee, and informed consent was waived. The patients enrolled in this study were selected from among all patients undergoing CESM at our institution between October 2011 and March 2013. The inclusion criteria were as follows: being female; being ≥ 18 years of age; having histologically proven primary breast cancer; and having received NAC as part of the treatment. Patients who had undergone surgical treatment other than lumpectomy or mastectomy were excluded, as were those for whom there were no results from the histological analysis of the surgical specimen. The precise regimen of NAC varied and was at the discretion of medical oncologist in charge.

### CESM examination

All CESM examinations were performed with a commercially available FFDM system (SenoDS/SenoBright; GE Healthcare, Buc, France). The dual-energy technique was applied under the supervision of a radiologist.

Dual-energy CESM exams were performed by acquiring a pair of low- and high-energy images during a single breast compression. Low-energy images were obtained with a molybdenum or rhodium target and filter, whereas highenergy images were acquired with a molybdenum or rhodium target and a copper filter. Both images were acquired with automatic optimization of parameters.

A 1-2 mL/kg dose of a nonionic contrast agent (iohexol, 300 mg/mL) was injected intravenously with an automated injector at a flow rate of 3 mL/s. Imaging was initiated 1.5-2 min after the injection and continued for 3-5 minutes. Bilateral craniocaudal and mediolateral oblique views were acquired. The complete examination protocol has been described and explained in detail elsewhere^(17)^.

It has been demonstrated that low-energy images are equivalent to FFDM, even in the presence of intravenous iodinated contrast^(18)^. In this study, we use the terms FFDM and CESM to refer to low-energy images and recombined images, respectively.

### Image analysis

Before the readings, a radiologist with three years of experience in CESM (reader 1) conducted a training session. Training cases were provided in order to familiarize the other radiologists with CESM and with the reading protocol. The same radiologist selected the cases for this study, split the low-energy and recombined images, anonymized them, and loaded them (separately and together) into a workstation.

A breast radiologist with over ten years of practice (reader 2) and a breast imaging fellow (reader 3) reviewed all studies using the same radiology workstation (Seno Advantage 2.2; GE Medical Systems, Milwaukee, WI, USA). Tumor laterality was the only background information available.

To avoid memory bias, the readings were conducted in two review sessions with a two-week interval between them. In the first session, only low-energy images were included, whereas low-energy and recombined images were included in the second review session. During the second session, the images were reviewed separately or together. Data from readers 2 and 3 were comparable to those in the original report submitted by reader 1.

### Pathology

Specimen processing was performed at a hospital, according to the protocols of the local institution. All pathological data were extracted from the pathology reports.

### Tumor size measurement

Suspicious findings were measured on low-energy and recombined images, in the craniocaudal and mediolateral oblique views. Some tumors presented as multiple enhancing spots, irregular masses, or ill-defined asymmetric masses. In those cases, the measurement included the largest tumor diameter

For analysis purposes, the largest diameters of the residual tumor documented on low-energy (FFDM) and recombined (CESM) images were compared with that determined for the pathological specimen.

### Statistical analysis

The size of the residual tumor determined by pathology was set as the "gold standard" and was compared to the size determined from the analysis of the low-energy and recombined images. The agreement between the size determined by pathology and that determined from the low-energy (FFDM) and recombined (CESM) images was assessed with the Bland-Altman 95% limits of agreement and intraclass correlation coefficient (ICC)^(19)^. Tumor size based on the FFDM and CESM images was also categorized as in agreement, underestimated, or overestimated, in relation to the size determined by pathology. We used scatter plots and Pearson's correlation coefficients to explore whether the size of the residual tumor determined by pathology correlated with that determined from the CESM and FFDM images. Values of *p* < 0.05 were considered significant. The interobserver agreement for each imaging technique was calculated using the limits of agreement and ICC. Statistical analysis was performed using MedCalc for Windows, version 14.8.1 (MedCalc Software, Mariakerke, Belgium).

## RESULTS

### Study population

We identified 12 lesions in 11 patients who met the inclusion criteria. Three patients (with a collective total of four lesions) were excluded because two died before surgery and the pathology result was not available for one. Therefore, the final sample comprised eight lesions in eight patients. The mean patient age was 46.41 ± 15.19 years (range, 22-76 years). The mean time from CESM and surgery was 32.6 ± 22.4 days (range, 5-66 days).

### Residual tumor size

**Figure 1:**
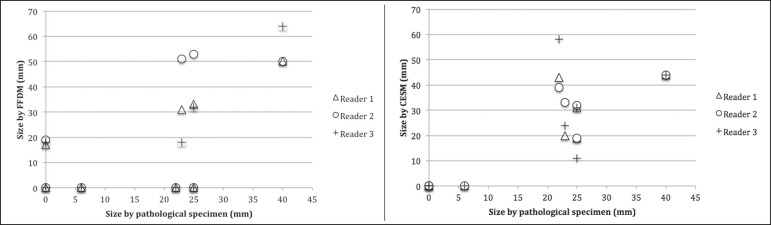
Scatter plot of the largest diameter of post-NAC residual breast tumors, as determined by pathology, from FFDM images, and from CESM images.

Residual tumor size ranged from microscopic (not measurable) to 40 mm, with a mean size of 17 mm. The size of the residual tumor determined from analysis of the pathology specimen is shown in Table 1, as are the sizes determined by all readers from the FFDM and CESM images. Scatter plots of those measurements are shown in Figure 1.

**Table 1:** Residual breast tumor size determined by pathology, from CESM images, and from FFDM images.

		Reader 1			Reader 2			Reader 3	
	Pathology	FFDM	CESM		FFDM	CESM		FFDM	CESM
Subject	(mm)	(mm)	(mm)		(mm)	(mm)		(mm)	(mm)
1	23	31	20		51	33		18	24
2	25	33	31		53	32		32	31
3	22	0	43		0	39		0	58
4	0	0	0		0	0		0	0
5	6	0	0		0	0		0	0
6	40	50	44		50	44		64	44
7	25	0	19		0	19		0	11
8	0	17	0		19	0		18	0

**Figure 2:**
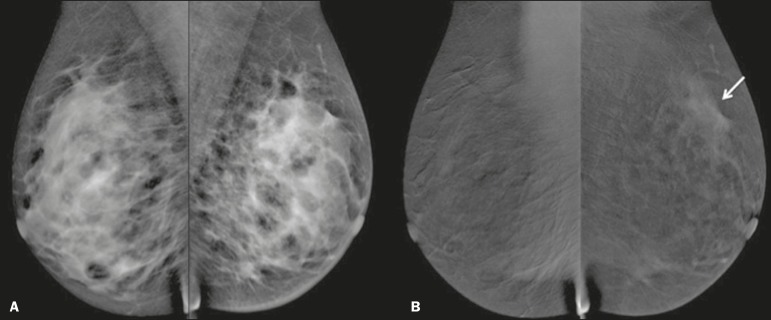
Residual (2.2-cm) tumor in a 23-year-old woman. FFDM (**A**) was negative, whereas CESM (**B**) overestimated the tumor size.

The pathological analysis revealed residual tumors in six (75%) of the eight patients evaluated. Three residual tumors were not visible on FFDM. Among those three tumors, CESM missed one, overestimated one, and underestimated one (Figure 2). CESM was true negative in both of the patients in whom the pathological analysis failed to identify a residual tumor, CESM also revealed no residual tumor at histopathology, whereas, in one patient, a focal asymmetry on FFDM was interpreted as a residual tumor by all readers.

**Figure 3:**
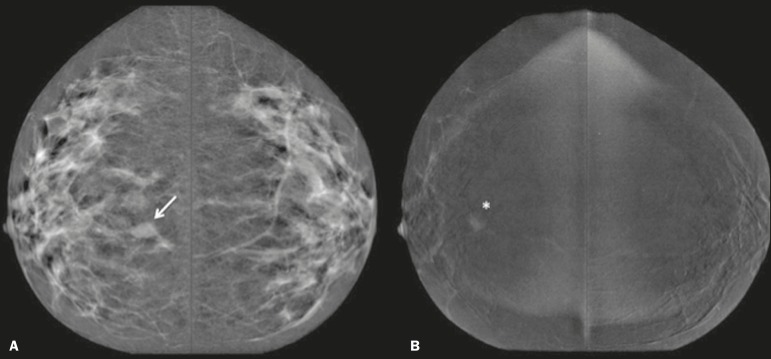
Residual (2.2-cm) tumor in a 45-year-old woman. On FFDM (**A**), all readers mistook a fibroadenoma for the tumor (arrow). On CESM (**B**), there was heterogeneous enhancement consistent with a residual tumor (asterisk).

One patient also had a fibroadenoma (Figure 3). On the basis of the FFDM images, all of the readers incorrectly held it as suspicious and concluded that the index tumor, which was located in the same breast, had been overrun. On CESM, the index tumor showed mild enhancement and the fibroadenoma showed none.

The sensitivity and specificity of CESM for detecting residual tumors were 83.33% and 100%, respectively, compared with only 50% (for both) for FFDM. A positive CESM examination was predictive of a residual tumor in 100% of the cases, twice as many as did a positive FFDM examination, whereas a negative CESM result predicted the absence of a residual tumor in 66%, compared with 25% for a negative FFDM result.

**Figure 4:**
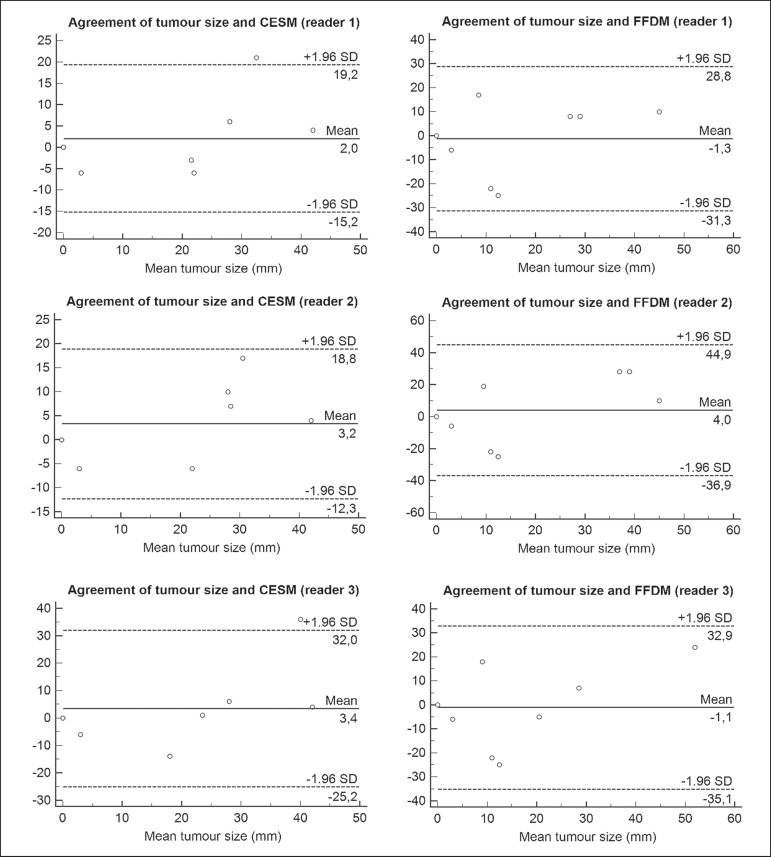
Bland-Altman analysis of the residual tumor size determined by pathology in comparison with that determined from the CESM and FFDM images, by all three readers.

Among all readers, the ICC between the size of the residual tumor determined by imaging and that determined by pathology was higher for CESM than for FFDM. The LoA was also better for CESM than for FFDM, as shown in Table 2 and Figure 4.

**Table 2:** Comparison among the three readers in terms of the mean residual breast tumor size as determined from CESM and FFDM images, as well as the limits of agreement, intraclass correlation coefficient, and correlation coefficient in comparison with the size determined by pathology.

	Reader 1			Reader 2			Reader 3	
Measure	FFDM	CESM		FFDM	CESM		FFDM	FFDM
Mean ± SD (mm)	-1.3 ± 15.33	2 ± 8.8		4 ± 20.8	3.25 ± 7.94		-1.1 ± 17.35	-1.1 ± 17.35
LoA (mm)	-31.3 to 28.8	-15.2 to 19.2		-36.6 to 44.9	-12.3 to 18.8		-35.1 to 32.9	-35.1 to 32.9
ICC (95% CI)	0.598 (0.11-0.90)	0.859 (0.45-0.97)		0.488 (0.26-0.87)	0.886 (0.53-0.97)		0.579 (0.14-0.90)	0.579 (0.14-0.90)
CC (*p*-value)	0.63 (0.094)	0.89 (0.003)		0.57 (0.138)	0.92 (0.001)		0.64 (0.086)	0.64 (0.086)

SD, standard deviation; LoA, limits of agreement; ICC, intraclass coefficient correlation; 95% CI, 95% confidence interval; CC, correlation coefficient.

Residual tumor size was underestimated by FFDM and CESM in 50% and 37.5% of the cases, respectively, overestimated by both in 37.5%, and correctly assessed in 12.5% and 25%, respectively.

There was a perfect agreement among readers regarding the presence or absence of residual tumor based on both FFDM and CESM (Table 1). The interobserver agreement was very good for both methods, although it was higher for CESM (Table 2).

## DISCUSSION

Recent studies in the radiology literature of Brazil have addressed the importance of imaging in the management of breast cancer^(20-27)^. Previous studies had corroborated the ability of CESM to detect primary breast tumors and demonstrated that its accuracy in preoperative tumor staging with lesion size measurement is comparable to that of MRI. In this feasibility study, we assessed the diagnostic performance of CESM in the detection and size determination of residual tumors after NAC in breast cancer patients.

Our findings make it clear that CESM is a feasible means of detecting residual tumors after NAC. In comparison with FFDM, CESM increased the sensitivity and specificity of residual tumor detection from 50% to 83.33% and from 50% to 100%, respectively. On post-NAC FFDM images, residual tumors were missed in three patients (37.5%), compared with only one (12.5%) on post-NAC CESM images. Our data also suggest that a positive CESM indicates the presence of residual tumor after NAC (positive predictive value, 100%).

Previous studies have demonstrated the greater accuracy of CESM in breast tumor measurement in comparison with MRI^(13,15)^, ultrasound^(16)^, and FFDM^(12)^. In the present study, we were able to show that the accuracy of CESM in residual tumor size determination was better than was that of FFDM, and both methods showed good agreement with the pathological findings (ICC = 0.692-0.886 and 0.488-0.598, respectively), although the correlation was significant only for CESM. The residual tumor size was overestimated less often by CESM than by FFDM. That might be especially meaningful in order to avoid unnecessary mastectomies and reduce the extent of breast conservation surgery.

Although interobserver agreement was very good for CESM and FFDM, it was slightly better for CESM. There was perfect agreement among readers regarding the presence or absence of residual tumors as determined by FFDM and CESM. The use of CESM increased the diagnostic performance of all readers, as was also reported by Dromain et al.^(28)^ in a study with six readers. Cheung et al.^(29)^ found that, among four blinded readers (radiologists) who scored lesions in terms of the probability of malignancy, interobserver agreement was significantly higher for CESM than for FFDM (0.62 vs. 0.38), whereas the difference between the two methods was smaller in the present study (0.94 vs. 0.88). Although this discrepancy is most likely attributable to a difference in sample size, it could also be because, in our study, the correlation was made on the basis of measurements, rather than scores, which are less subjective.

The present study has certain limitations, chief among which is the small size of the sample. The small sample size precluded an analysis of diverse cancer types and the collection of data regarding the influence of molecular subtypes. In addition, because CESM was not performed before or during NAC, we were unable to predict the response or estimate tumor size reduction. Readers were not totally blinded, because the laterality of the tumor was known to them. We were also unable to perform an analysis of the impact of CESM on surgical decision-making.

## CONCLUSION

Our results confirm that CESM is a feasible, easily performed method for evaluating residual tumor size after NAC. CESM correlates well and shows good agreement with the pathology, as well as showing good interobserver agreement. Therefore, our findings might be used as reference data for future prospective studies designed to evaluate the impact of CESM on surgical decision-making.
